# Aphid wing polyphenism and conspecific egg cannibalism affect the developmental and reproductive performance of *Chrysoperla carnea*

**DOI:** 10.3389/finsc.2025.1562606

**Published:** 2025-04-22

**Authors:** Ahmed A. Rashed, Marwa M. Ramadan, Mona M. Shalaby, Amged El-Harairy, Mohamed H. Bayoumy

**Affiliations:** ^1^ Economic Entomology Department, Faculty of Agriculture, Mansoura University, Mansoura, Egypt; ^2^ Department of Crop and Animal Sciences, Albrecht Daniel Thaer-Institute of Agricultural and Horticultural Sciences, Faculty of Life Sciences, Humboldt-Universität zu Berlin, Berlin, Germany; ^3^ Unit of Entomology, Plant Protection Department, Desert Research Center, Cairo, Egypt

**Keywords:** egg viability, ephestia, fecundity, non-aphid prey, survival

## Abstract

Aphid polyphenism and egg cannibalism may have nutritional consequences for the development, survival, and reproduction of predatory insects. Although predators have the same probability of attacking winged and wingless morphs in natural conditions, an increment in the proportion of winged morphs dispersed under predation risk may have a negative effect on predator feeding by reducing the size of the wingless form available on the plant. However, the wingless aphids may be richer in nutritional value than the dispersed winged aphids. Therefore, the nutritional consequences of aphid morphs and egg cannibalism for development, survival, 10-clutch fecundity and fertility, and the time needed for 10 clutches of eggs *Chrysoperla carnea* were addressed via a series of experiments. Wingless aphids accelerated the total development and increased the survival of *C. carnea* compared to the winged aphids. Furthermore, feeding with the wingless form increased the 10-clutch fecundities and fertilities, and reduced the days needed for 10 clutches of eggs. Neonate larvae of *C. carnea* that devoured two conspecific eggs took a shorter time with an acceleration in the overall development of *C. carnea*. Immature mortality was higher in controls than in the cannibalism treatment. Reproductive benefits were obvious in females permitted to consume two conspecific eggs during their first instar compared to those that did not. However, the time needed for 10 ovipositions did not differ between both groups. These findings are ecologically significant because *C. carnea* females are able to adapt to the stresses imposed by nature without needing winged aphid prey to distribute their eggs widely, as their larvae can grow on their own eggs and gain developmental and reproductive benefits from such behavior when prey availability or quality is low.

## Highlights

- Wingless aphids accelerated the development and increased survival of *C. carnea* compared to winged aphids.
- Feeding with wingless aphids increased female fecundity and fertility, and reduced days needed to obtain 10 clutches of eggs.
- Neonate larvae of *C. carnea* that cannibalized two conspecific eggs had faster development, higher survival, and their females gained more reproductive benefits than the controls.

## 1 Introduction

Aphid species are attacked by a large number of natural enemies that vary highly in the way they attack and their effectiveness on populations of aphids (1–3). Polymorphism is a general phenomenon in aphid species whereby female aphids give birth to genetically identical wingless and winged offspring during the asexual reproductive stage in cyclical parthenogenetic organisms (1, 4). This phenomenon seems to be a case of phenotypic plasticity; the mothers often perceive the environmental conditions and exert maternal impacts on the progeny (1, 5–7). In some cases, phenotypic plasticity expresses highly morphologically distinct outcomes and this is called wing polyphenism (8). Wingless and winged phenotypes vary as they invest either in reproduction or in dispersal (9, 10). In many aphid species, winged dispersal (alate virginoparae) morphs have been found to be produced in response to increased crowding and interspecific interactions, and to decreased quality of host plant and abiotic factors (1, 4, 6, 9, 10). Increasing crowding among individuals in an aphid colony generates significant tactile stimulation among individuals, which may lead to the production of wings (11).

More recently, it has been noted that the mere presence of predators and/or parasitoids has been known to induce the production of winged progenies (3, 12–14), e.g. in the cotton aphid, *Aphis gossypii* Glover (15) and the pea aphid, *Acyrthosiphon pisum* (Harris) (3, 5, 12, 13), but not in the Vetch aphid, *Megoura viciae* Buckton (13, 16). An advantage of an aphid colony is that it rapidly gives birth to winged forms under the threat of predation as a way to abandon the plant if the threat is large. Winged forms vary from wingless forms in a number of features, plus their ability to fly to invade and colonize new habitats. In several aphid species, wingless morphs have a shorter developmental time, a higher fecundity, and/or longer longevity than the winged morphs, which is likely due to the greater energetic cost of producing wings (1, 9, 17, 18). Although predators have the same probability level to attack both winged and wingless morphs under natural conditions, the increment in the proportion of winged morphs under threat of predation may have a negative effect on predators by reducing the colony size of the wingless aphids available on the plant for predator feeding. However, at the same time, the rest of the wingless aphids available for consumption may be richer in nutritional value than the dispersed winged morphs. The nutritional value of wingless females may correspond to a greater amount of embryos in the females, whereas the winged females have more musculature, sclerotized tissue, and wings, which do not contribute to the nutritional needs of developing lacewings. However, no previous study has yet systematically compared the mutual costs of wing formation on predator fitness in terms of development, survival, and reproduction. Thus, in this paper, the question arises: Are winged aphids similar to non-winged morphs in their qualitative effect on predator fitness or not?

Another factor that affects the population of predatory insects is cannibalism. The definition of cannibalism is a process in which individuals of the same species kill and eat each other, and it is a behavioral phenomenon that has been observed in a number of species. Cannibalism occurs during all life stages from immediately after hatching and throughout development or mating in many different social and ecological contexts (19, 20). Within the order of Neuroptera, cannibalism appears to be associated with polyphagous feeding habits (21). Chrysophid larvae are voracious and are commonly polyphagous feeders, thus their diet also involves prey of conspecifics (22). These larvae are active hunters, identified by fast movements, aggressive behavior, and rapid development. As shown by Wilson and Gutierrez (23) in California, their populations often consist of few larvae and many eggs and they expected that the population decline may be due to high mortality resulting from egg cannibalism. In the absence of aphids, 100% cannibalism among lacewing larvae was reported by Mochizuki et al. (24), but if aphid populations were abundant, the incidence of cannibalism was minimal. Likewise, when there is a lack of food under natural conditions, cannibalism is the only way for populations to survive as it is the only possible option to prevent local disappearance (25).

Cannibalism has been confirmed in a large number of living organisms, giving rise to several theories about its benefits and costs (19, 26, 27). Direct acquisition of nutrients and indirect exclusion of the same competitor individuals are among the most common gains of cannibalism. These gains are clearly context-dependent and their magnitude will differ depending on the nutritional status of the cannibal and the severity of cannibalism. Benefits can also differ depending on the developmental stage in which cannibalism appears. Since the physiological role of the larval stage is growth, we would speculate there are direct gains to larvae in terms of immature survival and development, and to adults in reproduction (e.g., 28). Aside from avoiding starvation, the only possible direct benefit for adults is reproduction, *i.e.*, increased egg quantity and/or offspring quality. However, few studies to date have attempted to estimate the relative direct and indirect impacts of cannibalism on life history plasticity and behavioral change at specific life stages (29).

When cannibalism is driven by the nutritional benefits, egg stage is usually the object (see 28 for relative cannibalism proportions in non-carnivorous insects at various life stages); they usually lack defenses, have high digestibility due to a lack of differentiation, and are rich in fats and proteins (30). Even species that in nature rarely tend to cannibalize their own eggs have succeeded in developing their own eggs (e.g., 31). For instance, the use of trophic eggs is common in eusocial insects (32). In many insects, neonates eat their own chorions following hatching, and then consume the unhatched eggs to scavenge the protein (e.g., 33). For instance, when predatory anthocorid bugs are hungry, they often prefer eating their own eggs compared to all other developmental stages (34). However, adults of phytoseiid mites prefer to eat their own larvae because their eggs are hard for them to penetrate (35). The egg stage of some prey species may have chemical defenses against heterospecific predators, whereas these are often ineffective against siblings (36).

Neonate larvae of coccinellid predators may raise their survival rates by preying upon sibling eggs (37, 38), while larval cannibalism increases rates of successful pupation and emergence (39). Thus, in adversity, cannibalism in coccinellids is adaptively significant to raise their survival rates (40). For example, Bayoumy and Michaud (38) discovered the life history benefits and costs of egg cannibalism in the youngest and oldest larval instars and adult stages of *Hippodamia convergens* Guerin-Meneville. By the neonate larvae stage, it had increased the larval development rate and male adult size, while egg cannibalism by the adults had no effect on their fecundity or fertility. In contrast to the previous study, Abdelwahab et al. (41) reported no benefits for neonate egg cannibalism on development and reproduction in *Coleomegilla maculata* DeGeer. However, the egg cannibalism consequences for the developmental and reproductive performance of *C*. *carnea* have not yet been discovered.

This study therefore aims to clarify the nutritional costs of larval feeding on winged aphids or conspecific eggs for the development and reproduction of *C. carnea*. The generalist species, which have the same probability level to attack both winged and wingless morphs under natural conditions, few of which would present a hazardous environment to an aphid colony, was hypothesized to respond with stress to the presence of winged aphids, resulting in a phenotype with reduced fitness relative to the wingless form, and we hypothesize that larval egg cannibalism would yield measurable benefits for the development performance and reproductive success of *C. carnae*. These relationships are of ecological importance as they help us understand the extent to which predators can adapt to conditions of limited natural prey, whether cannibalism is a way to overcome this, and whether the predator’s larvae are able to adapt to the conditions imposed by nature by increasing the proportion of winged aphids in the aphid colony without affecting its reproduction.

## 2 Materials and methods

### 2.1 Aphid colony

Seeds of broad bean (*Vicia faba* L.) were cultivated in plastic pots (3.0 cm in diam.) with sand, peat moss, and perlite (1:1:1). These pots were maintained under normal room conditions. The seedlings were irrigated daily until they reached 3–4 cm in tall. Once the seedlings attained the appropriate tall for artificial infestation, the cowpea aphid, *Aphis craccivora* Koch was moved to these seedlings. Broad bean plants heavily infested with *A. craccivora* were obtained from the Biological Control Insectary at Cairo University as a source of aphids. These plants were in contact with the pots containing clean broad bean plants by cutting off parts of the infested plants and attaching them to the new bean plants. The aphid culture was maintained under room conditions (26 ± 1 °C and 60 ± 5% RH) and the colony was provided with normal light during daylight hours using metal halide lamps. The aphids were settled on the upper portion of the plant materials at the first four internodes.

### 2.2 Induction of winged aphid morphs

The proportion of winged dispersal morphs of cowpea aphids can be increased in the presence of ladybeetles or parasitoids (3, 12, 13). Accordingly, the winged morph of cowpea aphid was induced either by placing some infested pots in dark conditions or by keeping a couple of touching Petri dishes (9.0 cm in diam.) separated by internal holes (to permit physical odors of ladybirds to create a fear response), with one having high numbers of aphids with a small piece of a broad bean plant and the second having at least 10 eleven-spotted ladybird *Coccinella undecimpunctata* adults in an incubator set to 27.0 ± 1.0°C. The plants were changed as required. The winged morphs of *A. craccivora* were separated daily for consumption experiments. The proportion of winged dispersal morphs of cowpea aphids was close to 25%. However, the proportion was higher under conditions of darkness than those of threat by natural enemies.

### 2.3 Predator culture

The culture of *Chrysoperla carnea* (Stephens) (Chrysopidae: Neuroptera) was started with 60 adult individuals (1-day old) obtained from Beneficial Insectary, Cairo University. Once the shipment arrived, lacewings were separated into two clear plastic jars (each 20 × 40 × 8 cm). The jar opening was closed with a black mesh screen using a rubber band that was attached in place. Then, these containers were kept at normal room conditions (26.0 ± 1.0°C, 60 ± 5% RH, and 14L:10D photoperiod). In each jar, predator adults were provided with two sponges saturated with a mixture of honey and brewer’s yeast and another one saturated with water, both renewed every 2 days. The eggs laid on a black screen were collected daily by snipping their stalks with scissors into 9.0 cm Petri dishes. After hatching, larvae were reared individually in 5.5 cm Petri-dishes to prevent cannibalism. All nutritional sources were strictly controlled to ensure they came exclusively from the designated aphid morphs.

### 2.4 Influence of aphid morphs on the development and reproduction of *Chrysoperla carnea*

In this trial, the eggs collected from the first *C. carnea* female generation were collected in 5.5 cm Petri dishes and maintained at 25.0 ± 1.0°C, 60 ± 10% RH, and a 16:8h L:D photoperiod until hatching. In these conditions, the incubation period ranged between 3–4 days. All larvae used in the trial were isolated on the same day of emergence and therefore their incubation period was fixed (*i.e*., 4 days at the physical conditions that were previously described). The first instar larvae of *C. carnea* were divided into two groups. The first group (60 larvae) was provided daily with winged morphs (petrous form) of *A. craccivora*, whereas the second one (60 larvae) was supplied with the wingless form (apterous form) of cowpea aphid. The number of adult aphids of both forms that were provided daily for each individual predator differed according to the larval instar (*i.e*. larval size). Accordingly, the first instar was provided with 10 aphids, the second with 15 aphids, and the third instar with 20 adult aphids. Aphids were introduced to each larva in a 5.50 cm Petri dish on white filter paper fixed in the bottom of the dish. Every day, the Petri dishes were swabbed and the larvae were supplied with fresh aphids of each morph, regardless of whether they were consumed or not. Larvae were monitored every 12 h to record the larval, pupal, and total development times. The number of larvae that (immature survival) completed their development was recorded. Furthermore, lacewings that emerged successfully, but died immediately after emergence or those that emerged incompletely from the pupae and did not have the ability to fly (i.e. malformed) were considered dead.

Once the progeny of the parents emerged, all the lacewings that emerged on the same day of each group were gathered in one jar (30 cm × 50 cm × 10 cm) and left for 4 days to confirm mating. The neck of these jars was handled as previously described. Four days later, the adults were sexually isolated. Female lacewings were isolated in a jar (10 cm × 20 cm × 5 cm), marked with a number indicating their individual identity, and supplied daily with drops of honey and yeast, as previously described, until each female laid eggs on 10 different days. Roughly, most females began laying their eggs on the fifth to sixth day. Each group started with 12 females, but females that failed to produce eggs on 10 different days were not considered in the analysis. The eggs laid by each female were collected daily in a 5.5 cm Petri dish and kept in an incubator (25.0 ± 1.0°C and a photoperiod of 16L:8D). Some of the frozen eggs, *Ephestia kuehniella*, were provided in each dish to prevent cannibalization of eggs by earlier hatching larvae. Three days later, the hatching rate for each female in each treatment was estimated. Life history data involving time to pupal and adult stages, and larval and pupal survivals were considered. The total fecundity and fertility and time needed for 10 ovipositions in each female were recorded.

### 2.5 Influence of egg cannibalism on the development and reproduction of *Chrysoperla carnea*

In this experiment, two sets of newly emerged larvae (n = 30 per set) were separated into 5.5 cm Petri dishes within 24 h of hatching to compare the egg cannibalism consequences for the development of *C*. *carnea*. The first set of *C. carnea* larvae, in isolation, was supplied with two sibling eggs (<24 h old) in their first day of life, while those of the second (control) were supplied only with the frozen eggs of Ephestia. Both groups were provided with water via a small sponge. A previous study has demonstrated that consuming one egg, in the first instar, can lead to great change in the life history of the larval stage (42). Once all the larvae of the first group cannibalized their sibling eggs (<6 h), they were then offered *ad libitum* Ephestia eggs and water, both refreshed daily. The larvae of both groups were provisioned daily with Ephestia eggs until they pupated. These larvae were checked every 12 h so that all transitions in development could be counted to the nearest day. On the same day of adult emergence, all adults of each group were collected in a jar (30 cm in diam. × 50 cm in ht.) to ensure mating. The jar opening was closed with black muslin attached in place with a rubber band. These jars were maintained at 25.0 ± 1.0°C, 60 ± 10% RH, and 16L:8D photoperiod. The adults in the jars were fed with a diet and water as previously described. Six days after emergence, females began to lay eggs. Once eggs were observed, the females from each group (n = 9) were isolated in small jars (10 cm in diam. and 20 cm in ht.) and feeding and physical conditions were followed as previously described. Eggs were collected 10 times of eggs from each female. The laid eggs were collected, handled, and kept in the same physical conditions as previously described. A few frozen Ephestia eggs were provided in each dish to prevent cannibalization of unhatched eggs by earlier hatching larvae. Three days later, the hatching rate for each female in each treatment was recorded. Datasets, including time to reach the pupal and adult stages and larval and pupal survival, were considered. The total fecundity and fertility and time needed for 10 clutches of eggs were calculated.

### 2.6 Data analysis

Datasets on development and reproduction for the green lacewings were analyzed for planned comparisons (either winged vs. wingless aphids or cannibals vs. non-cannibals) using an independent *t*-test. To address the relevance of egg cannibalism more directly, ANOVA was performed. Before using ANOVA, the data were inspected for normality (Shapiro–Wilk test) and equality of variance (Levine’s test). Because the normality test failed (P > 0.001) for the development and fecundity data, a one-way ANOVA on ranks was implemented. In contrast, the fertility data passed both tests (P < 0.05) and thus one-way ANOVA was applied. Furthermore, the development and reproduction data of the green lacewings were tested using ANOVA between the first instar larvae that fed on winged aphid morphs, wingless aphid morphs, and conspecific eggs. *Post hoc* comparisons were conducted using Dunn’s test for non-parametric data (development, fecundity, and days required to obtain 10 ovipositions) and Tukey’s test for normally distributed data (egg fertility). These tests were selected based on data distribution and sample size characteristics of each variable. Given the continuous nature of the developmental and reproductive data and the need to compare means rather than frequencies or proportions, the Chi-square test would not be suitable for most comparisons and the proportions of progeny that succeeded in completing their development to adult emergence were compared between each pair of groups using Fisher’s exact test (43).

## 3 Results

### 3.1 Influence of aphid morphs on the development and reproduction of *Chrysoperla carnea*

The larval stage duration of *C. carnea* was significantly shorter for those fed the wingless form of aphid than those provided with the winged form. However, the pupal stage duration did not differ between both groups of aphids. The total developmental time of *C. carnea* differed between larvae fed daily with the wingless form of aphids and those fed the winged form. Further, the survival percentage was significantly higher for lacewings that were provided wingless aphids than those that received winged aphids (**Table 1**).

**Table 1:** Effect of aphid wing polyphenism on developmental parameters (± SE) of the progeny of *Chrysoperla carnea*, in which the larval stage was fed daily a diet of wingless or winged aphids, *Aphis craccivora*.

Biological parameter	Aphid form		Test	
	Wingless	Winged	*t*	*p*
Egg (days)	4.00 ± 0.00	4.00 ± 0.00	NT	NT
Larval (days)	14.31 ± 0.65	18.44 ± 0.44	5.29	< 0.001
Pupal (days)	9.24 ± 0.13	9.48 ± 0.12	1.31	0.19
Total development (days)	27.55 ± 0.67	31.92 ± 0.46	5.40	< 0.001
Survival %	84.00	74.00	Fisher	< 0.001

NT, Not statistically tested.

**Figure 1:**
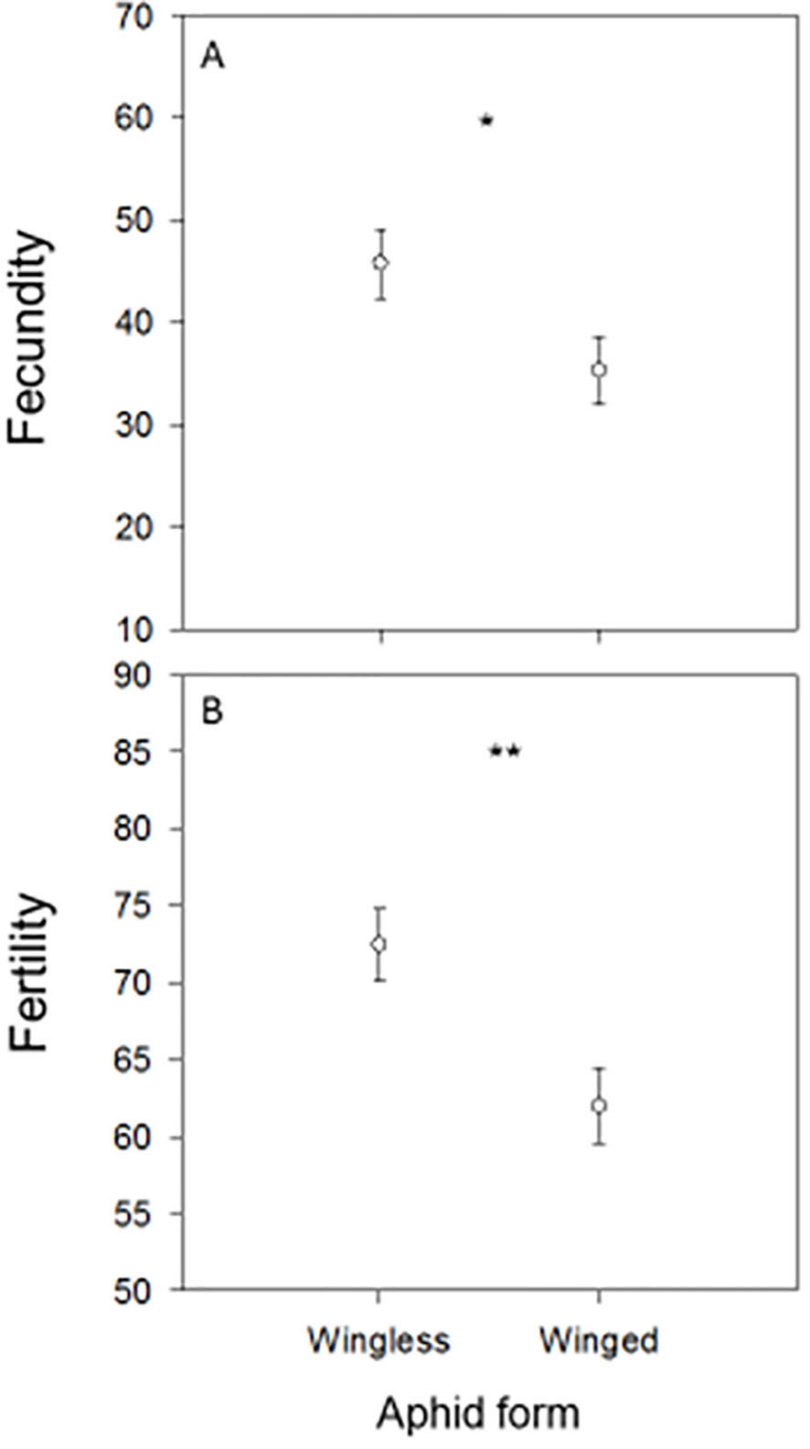
Mean (± SE) 10-clutch egg fecundities **(A)** and egg fertilities **(B)** of *Chrysoperla carnea* females that emerged from progenies fed in their larval stage on the winged or wingless form of the cowpea aphid, *Aphis craccivora*. * and ** denote *P* < 0.05 and *P* < 0.01.

The independent *t*-test demonstrated that there were significant variations in 10-clutch fecundity between females that were fed wingless aphids as larvae and those that were fed winged aphids (*t* = 2.20; *P* = 0.04), as well as in egg viability (*t* = 3.05; *P* = 0.007) and in the days required to obtain ten clutches of eggs (*t* = 0.78; *P* = 0.45). The 10-clutch fecundity was higher in females fed wingless aphids (45.77 ± 3.39 eggs) than those that received winged aphids (35.30 ± 3.32 eggs). Furthermore, the 10-clutch egg viability rate (*i.e*. fertility rate) was higher in females fed wingless aphids (0.73 ± 0.02) than those that received winged aphids (0.62 ± 0.02). Females that as larvae were fed wingless aphids laid their 10 clutches of eggs in a shorter period (14.56 ± 1.39 days) than those fed winged aphids (16.60 ± 2.16). The *F* test result between the wingless and winged parameters was 1.35 with significant a *P*-value (**Figure 1**).

### 3.2 Influence of egg cannibalism on development and reproduction of *Chrysoperla carnea*

Neonate larvae of *C. carnea* that consumed two conspecific eggs had a shorter time in the larval stage than those in control and eventually obtained faster overall development, despite no influence of cannibalism on pupal stage duration (**Table 2**). Mortality (larval-adult) was significantly higher in controls (13.33%) than in the cannibalism treatment (3.33%).

**Table 2:** Effect of egg cannibalism on developmental parameters (± SE) of neonate larvae of *Chrysoperla carnea* that were allowed to consume two sibling eggs (<24 h old) on the first day of their life (cannibals) compared to those that did not (control).

Biological parameter	Diet		Test	
	Control	Cannibals	*t*	*p*
Egg (days)	4.00 ± 0.00	4.00 ± 0.00	NT	NT
Larval (days)	14.64 ± 0.14	13.55 ± 0.27	3.51	< 0.001
Pupal (days)	10.34 ± 0.31	9.59 ± 0.18	2.06	0.05
Total development (days)	29.00 ± 0.36	27.14 ± 0.25	4.38	< 0.001
Survival %	86.67	96.67	Fisher	< 0.001

NT, Not statistically tested.

**Figure 2:**
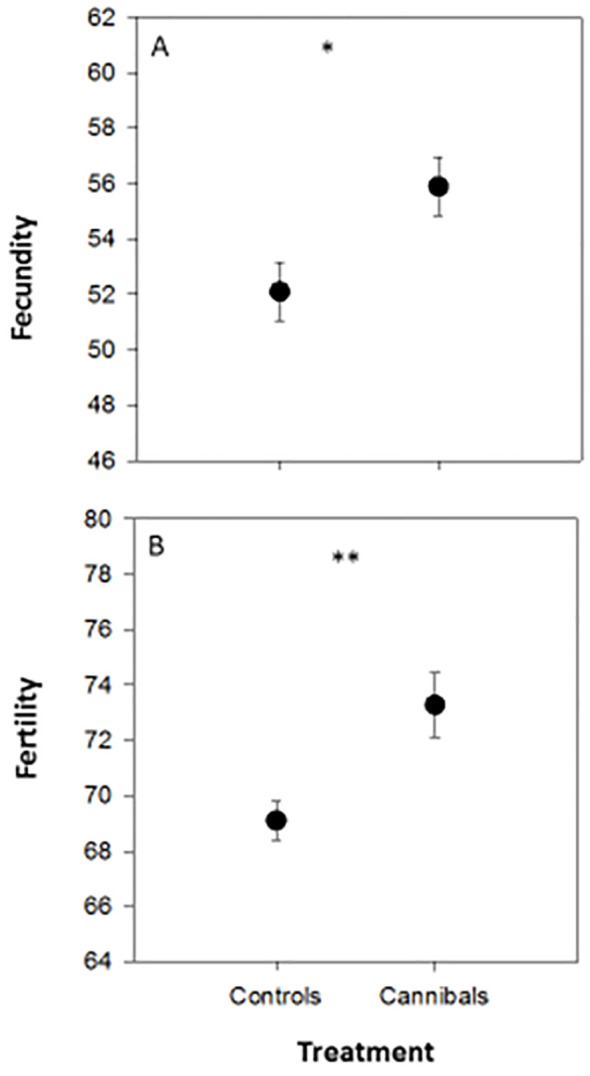
Mean (± SE) 10-clutch egg fecundity **(A)** and egg fertility **(B)** of *Chrysoperla carnea* females that their first instar larvae were permitted to consume two conspecific eggs (<24 h old) within 24 h of exclusion (cannibals) compared to those did not (control). * and ** denote *P* < 0.05 and *P* < 0.01.

The independent *t*-test demonstrated that there were significant differences in 10-clutch fecundity between *C. carnea* females allowed to feed on two sibling eggs (<24 h old) during their first instar (cannibals) compared to those did not (control) (*t* = 2.59; *P* = 0.02), as well as in egg viability (*t* = 3.07; *P* = 0.006), but not in days required to obtain ten clutches of eggs (*t* = 0.40; *P* = 0.69). The 10-clutch fecundity was higher in females that cannibalized their own eggs in the first instar larvae (55.90 ± 1.04 eggs) than those that did not cannibalize (52.10 ± 1.03 eggs). Moreover, the 10-clutch egg viability rate (*i.e.* fertility rate) was higher in females that cannibalized their own eggs in the first instar larvae (0.73 ± 0.12) than those that did not cannibalize (0.69 ± 0.07). Females that cannibalized their own eggs in the first instar larvae laid their ten clutches of eggs in a shorter period (14.20 ± 0.43 days) than those that did not cannibalize their own eggs (14.50 ± 0.52). The *F* test result for the effect of egg cannibalism on developmental parameters was 1.26 with a significant *P*-value (**Figure 2**).

ANOVA revealed that green lacewings that were fed with two conspecific eggs during their first instar had the fastest development (*H* = 25.96, df = 2, *P* < 0.001), the highest fecundity (*H* = 14.85, df = 2, *P* < 0.001), and the greatest fertility (*F*_2,28_ = 9.51, *P* < 0.001) compared to those fed with wingless or winged aphids. Furthermore, there were no significant differences between the females obtained from the three feeding groups in the days required to obtain 10 ovipositions (*H* = 0.29, df = 2, *P* = 0.87) (**Table 3**).

**Table 3:** Median (25%-75%) and ^†^mean (± SE) of development, 10-clutch egg fecundity and egg fertility, and days required for 10 ovipositions in *Chrysoperla carnea* that were permitted to feed on one of three diets (wingless and winged aphids, and conspecific eggs) as first instar larvae.

Biological parameter	Diet		
	Wingless aphids	Winged aphids	Conspecific eggs
Development (days)	24.00 b (20.0-28.0)	27.00 a (29.0-29.0)	23.00 c (22.0-24.0)
Fecundity (eggs/female/10 clutches)	43.00 ab (38.5-53.5)	33.00 b (25.5-45.25)	56.00 a (52.0-59.0)
^†^Fertility (% hatching)	72.5 ± 2.39 a	62.0 ± 2.45 b	73.3 ± 1.16 a
Days to obtain 10 ovipositions	13.00 a (11.0-19.0)	14.00 a (11.0-22.0)	14.00 a (13.0-15.5)

Values followed with the different letters are significantly different (ANOVA followed by Dunn’s or ^†^Tukey’s test).

## 4 Discussion

The present study examined whether aphid wing polymorphism and cannibalism have consequences for predator populations or not. Cannibalism is known to be a major constraint on mass production of *C. carnea* and increases its difficulties, requiring measures related to nutrition and rearing units. However, laboratory conditions such as lighting, temperature, low host plant quality, aphid crowding, and/or aphid deference responses may induce the production of winged morphs of aphids in the rearing units which used further for feeding those predators (e.g. 1, 6, 44). This may have some negative consequences on the growth and reproduction of the predator in the rearing units. Thus, both factors are linked and may affect population dynamics of *C. carnea* under rearing conditions and then the number of predators required in inundative releases. To our knowledge, there are no available studies on the consequences of feeding on winged aphids to explain the current results. Larval feeding of *C. carnea* on wingless aphid morphs accelerated development and increased survival of *C. carnea* compared with winged morphs. In a large number of aphid species, the wingless morph has a shorter developmental time, a higher fecundity, and/or longer longevity than the winged morph, which is most likely due to the increased energy cost of having wings (1, 17, 18). The nutritional state of the prey may be a significant signal for assessing prey quality (45). The nutritional value of wingless females may correspond to the greater amount of embryos in the females, whereas the winged females have more musculature and sclerotized tissue and wings, which probably do not contribute to the nutritional needs of developing lacewings. The total development time of lacewings was significantly shorter in those fed with the wingless aphid form than those fed with the winged form. Demmon et al. (46) reported that the development of *Aphidius ervi* Haliday parasitoids in immature wingless forms had a positive effect on the size of emerging parasitoids. In a preference test for winged and wingless forms of *Aphis fabae* (Scopoli) and *Myzus persicae* (Sulzer) in *Coccinella septempunctata* (L.), the Manly’s preference index showed that the wingless form was consumed more significantly by *C. septempunctata* than the winged form (47). Thus, the preference for a particular species of prey may mean that predators feed on a particular species of prey independently of their abundance or accessibility (48). Moreover, predators feed on different types of prey available so as to maximize the nutritional gain while minimizing the costs and risks associated with predation, thus, when a predator encounters two types of prey, it selects the one most likely to maximize its net energy gain (49). The phenomenon by which predators seek, locate, and recognize suitable and palatable prey is still unknown (47).

The data revealed that the 10-clutch fecundity and fertility were higher in females that as larvae were fed wingless aphids than those that as larvae were provided with winged aphids. Moreover, the former laid their ten clutches of eggs in a shorter period than those fed winged aphids as larvae. Feeding on apterous virginoparous nymphs of *M. persicae* by *H. convergens* females resulted in higher fecundity than in the case of feeding on the same weight of alatiform gynoparous (50). The influence of the nutritional state of winged and wingless aphid morphs on the parasitoid’s fitness was compared by Pirotte et al. (51). They found that the third and fourth wingless aphid instars were higher in lipid, free sugar, and glycogen content. This may explain the reproductive benefits gained via the accumulation of resources by *C. carnea* larvae since the adults are not predacious and feed on other foods, such as pollen, nectar, and honeydew. Host fats and proteins are necessary for parasitoids to form eggs in the ovary (51, 52). However, the reproductive benefits of winged aphids for emerging parasitoids and the corresponding costs for lacewing females can be interpreted from an ecological perspective, as the endoparasitoids inhibit wing formation in the host (e.g., 46, 53) to re-orient resources that would not be available for their own growth (46), while chrysopid larvae prey on their prey by sucking body juice that is poor in nutrients that were previously directed to the formation of prey’s wings. In addition, wingless morphs may lay more offspring than winged morphs (54). Thus, such a reproductive advantage may indirectly contribute to an increase in the reproductive fitness of the female via increasing her body size and hence her fecundity.

Most chrysopid females do not have the ability to preclude egg laying in the presence of eggs of the same species, i.e., conspecific eggs, and the newly hatched larvae do not feed preferentially on eggs of non-relatives (heterospecific cannibalism) rather than those of their own relatives (conspecific cannibalism) (55). From the point of view of genetic relatedness, eggs produced by a female in an already used site are not more likely to be the victim of cannibals than those that are that already exist. Lacewing eggs become less susceptible to consumption by siblings as they become older. This suggests that the egg cannibalism risk by neonate larvae of the lacewing in nature may be high for newly laid eggs or for pre-hatching eggs, but may be lower in between these two events (55, 56). On the plant substrate, the eggs of lacewings, whether conspecific or heterospecific, would be therefore highly vulnerable to neonate cannibalism for a short period of their development, regardless of prey availability (57). Because of the conspecific (cannibalism) and heterospecific (intraguild) predation risks within aphid colonies, predators often lay their eggs at a certain distance that can be decided by the trade-off between the risks of starvation and predation of the newly hatched larvae (58). Mothers of ladybird predators lay their eggs in clutches to encourage their progeny to cannibalize their eggs, and lay some of these eggs, as infertile eggs, externally in the form of a ring. This behavior primarily provides the opportunity for neonates to remain alive in the field until they catch their first prey (59, 60). All of this information regarding the cannibalistic behavior of *C. carnea* encouraged us to examine the life history and reproductive costs and/or benefits of cannibalism. Egg cannibalism by *C. carnea* neonates accelerated development overall, consistent with findings for other species studied in this aspect (38, 42, 57, 61, 62) and inconsistent with others (e.g., 37, 41, 63). Noppe et al. (57) noted that the larvae of *C. carnea* exhibited greater adaptation to cannibalism than those of *C. maculata*, incurring fewer costs due to the behavior. Michaud and Grant (64) examined the benefits of neonate egg cannibalism on some biological aspects in three aphidophagous ladybeetles and found that this behavior accelerated development in all species, but had different effects on their body size (larger body size in either male, female, or both sexes). In another study, Pervez et al. (65) showed the cost of larval cannibalism by coccinellids on adult body weight. Immature survival (larval-adult) of *C. carnea* was significantly higher in the cannibal treatment than in non-cannibal treatment. Egg cannibalism is a way to keep the survival of neonate larvae if food in a short supply (e.g., 19, 59). The highest survival rates for larvae fed with their fresh own eggs compared to those fed with frozen *Ephestia* eggs can be ascribed to the high viability of eggs instead of cannibalism rates. In many insect species, neonates feed on their chorions after hatching to obtain the protein and lipids (e.g., 30, 33) which are important for growth, survival, and reproduction. Although eggs of certain prey species may exhibit chemical defenses against predators of other species, they are usually useless against siblings (36).

Larval egg cannibalism yielded apparent reproductive benefits in terms of higher fecundity and fertility and a shorter period to lay 10 clutches of eggs. Although we did not account for pupal and adult weights, the obtained benefits may be due to increasing the fresh body weights of emerging adults as reported in other reports (e.g., 38, 66, 67). Xin-Lan et al. (67) reported that consuming a small number of non-kin eggs over larval instars has minimal impact on the reproductive fitness of *H. axyridis* females, but consuming a large number of such eggs only in the last instar can significantly reduce the fecundity of adult females. However, giving *H. convergens* beetles the opportunity to feed on sibling eggs during their fourth instar led to faster pupation and higher production of eggs that hatched faster than those did not feed (38). These findings are consistent with those in more aggressive aphidophagous predators whose larvae acquired developmental benefits from egg cannibalism that perhaps extended to reproductive benefits.

The tendency to feed on both aphid forms is imposed on green lacewing larvae in the same aphid colony, especially in case of prey scarcity or competition with other species, while the tendency for egg cannibalism is an optional behavior and is due to the species itself even if there is prey in high numbers. The benefits acquired by neonate cannibals were verified by comparing development and reproductive outcomes of green lacewings that were permitted during their first instar to feed aphid morphs or to cannibalize their conspecific eggs. Comparisons between aphid wing polyphenism and egg cannibalism revealed that the latter factor is more important and contributed more to accelerating development and increasing female reproductively. The presence of significance between the reproductive outcomes of females reared during their larval stage on winged aphids and conspecific eggs and the absence of significance between females fed with wingless aphids and conspecific eggs in the same parameters clearly indicate a lack of resources (e.g. protein, lipids, and carbohydrates) necessary for growth and reproduction in winged aphid form which is likely due to the investment in flight attributes (68). This may affect the body size of the emerging adults and thus their reproduction especially since their reproduction mainly depends on the larval nutrition (69, 70). Female *C. carnea* require aphids to distribute their eggs widely in the habitat, since the larvae need aphid prey to develop. However, female *C. carnea* do not hesitate to oviposit in the presence of conspecific eggs to interfere with conspecific females. Therefore, *C. carnea* is subject to the intraspecific competition that selects for cannibalism in the more strictly aphidophagous species. In addition, given the very crowded structure of aphid colonies, intensive aphidophagy behavior may lead to significant levels of competition among conspecific larvae of *C. carnea*. These conditions can lead to cannibalism even if the nutritional advantages of such behavior are small, where it increases competition among larvae of the same species. As cannibalism has become an increasingly important factor in determining the fitness of species, other adaptations that enhance the benefits will be selected for (e.g., the most efficient use of the gained nutritional resources in development and reproduction) and costs minimized (e.g., filial egg recognition). This ecological inference is based on the strong tendency of the larval stages of lacewings to cannibalize each other (21, 57).

## 5 Conclusion

In summary, this study concludes that *C. carnea* females are able to adapt to the stresses imposed by nature without needing winged aphid prey to distribute their eggs widely, as their larvae can grow on their own eggs and gain nutritional benefits from such behavior when prey availability or quality is low.

## Data Availability

The original contributions presented in the study are included in the article/supplementary material. Further inquiries can be directed to the corresponding authors.
